# Anomalous Cilioretinal Artery With Associated Macroaneurysms: A Case Report and Literature Review

**DOI:** 10.7759/cureus.117184

**Published:** 2026-09-29

**Authors:** Abdulrahman Khan, Nada A Alyousef, Hanan A Alshalan, Abdulrahman H Badawi, Moustafa S Magliyah, Wael A Alsakran

**Affiliations:** 1 Vitreoretinal Division, King Khaled Eye Specialist Hospital, Riyadh, SAU; 2 Department of Ophthalmology, King Khaled Eye Specialist Hospital, Riyadh, SAU

**Keywords:** anomalous cilioretinal artery, hypertension, intravitreal anti-vegf, laser photocoagulation, vascular macroaneurysms

## Abstract

We present a rare case of multiple vascular macroaneurysms involving an anomalous cilioretinal artery in a hypertensive individual. A 42-year-old female presented with decreased vision in her left eye. Fundus examination and fluorescein angiography revealed bilateral Grade 2 hypertensive vasculopathic changes according to the Keith-Wagener-Barker classification, along with an anomalous cilioretinal artery characterized by an extensive branching network, extension to the far superotemporal quadrant, and macroaneurysms along its course, one of which was actively leaking with secondary macular lipid exudation in the left eye. Systemic evaluation revealed high blood pressure, and she was diagnosed with systemic hypertension and started on systemic antihypertensive medication. Treatment of the leaking macroaneurysm with focal laser photocoagulation was performed, in addition to peripheral sectoral laser, covering the ischemic temporal retina. Three intravitreal bevacizumab injections were administered without subject or objective improvement. Anomalous cilioretinal arteries associated with macroaneurysms are exceedingly rare. Our case illustrates that hypertensive vasculopathy, along with altered hemodynamic stress and overload on the abnormal vessel, may elevate the risk of macroaneurysm formation.

## Introduction

The cilioretinal artery is the most prevalent anatomical variation of the retinal circulation. It originates from the posterior ciliary artery, which provides further blood supply to the retina, primarily to the macular area [1,2]. The cilioretinal artery has been observed in up to 49.5% of individuals, with bilateral involvement reported in a subset of cases [3]. Although the cilioretinal artery typically supplies the papillomacular region, preserving central vision, anatomical variants and anomalies have been reported in which it supplies a substantially larger retinal territory, including half or, rarely, nearly the entire retina [2,4-6].

Retinal arterial macroaneurysms are acquired fusiform dilations that usually involve the first three orders of the retinal arteries in predisposed, advanced-age individuals with underlying atherosclerotic disease [7]. They may cause multilayered retinal hemorrhages, intraretinal fluid, and lipid exudation, particularly when located near the macular area [7]. However, vascular macroaneurysms involving the cilioretinal artery have been reported, albeit infrequently [8,9].

In this report, we present the case of a middle-aged hypertensive woman with multiple unilateral retinal arterial macroaneurysms arising along an anomalously elongated and extensively branching cilioretinal artery, associated with marked macular exudation and peripheral retinal capillary non-perfusion. We describe the clinical features, multimodal imaging findings, management outcome, and the longitudinal course of this unusual presentation.

## Case presentation

A 42-year-old female, not known to have any systemic medical illness, presented to the retina clinic complaining of blurry vision in the left eye for the past six months. Her past ocular history was unremarkable. On examination, her best-corrected visual acuity was 20/30 in the right eye and 4/200 in the left. Intraocular pressure (IOP) measured with a Goldman applanation tonometer was 15 mmHg in the right eye and 17 mmHg in the left eye. Slit-lamp examination indicated a clear anterior segment in both eyes. Dilated fundus examination (DFE) of the right eye revealed clear vitreous, normal optic nerve appearance, and attached retina, with sclerotic arteries and arteriovenous (AV) nicking, consistent with Grade 2 hypertensive retinopathy per the Keith-Wagener-Barker classification (Figure 1). DFE of the left eye showed clear vitreous, a healthy optic disc, dense macular exudates involving the fovea, an abnormally large cilioretinal vessel arising from the temporal edge of the optic disc, with extensive bifurcations and AV crossings, coursing toward the far superotemporal periphery, and three macroaneurysms along the anomalous cilioretinal artery, one of which is located at the site of AV crossing (Figure 2). Optical coherence tomography (OCT) of the macula demonstrated a normal appearance in the right eye. Macular OCT of the left eye indicated multiple hyperreflective lesions along the outer retina, corresponding to hard exudates, as well as intraretinal fluid (Figure 3). Wide-field fundus fluorescein angiography (FFA) of the left eye revealed early complete filling of the anomalous vessel preceding the central retinal artery, suggesting that it is a cilioretinal artery. Additionally, three aneurysmal dilations along the anomalous cilioretinal artery of the left eye were noted with progressive leakage that was more pronounced in only one of them. Peripheral temporal areas of capillary non-perfusion with secondary vascular remodeling were also noted (Figure 2). FFA of the right eye was unremarkable, with no evidence of a cilioretinal artery, exudation, or fluorescein leakage. Collectively, the early fluorescein filling confirming cilioretinal origin, the unusually elongated and extensively branching arterial course, and the confinement of multiple macroaneurysms to this vascular distribution supported the diagnosis of multiple retinal arterial macroaneurysms arising along an anomalous cilioretinal artery. The patient underwent systemic evaluation, which showed normal vital signs except for an elevated blood pressure, averaging 180/110 mmHg. Laboratory workup including glycosylated hemoglobin, lipid profile, and evaluation of hypercoagulable, atherosclerotic, infectious, and inflammatory etiologies was all unremarkable.

**Figure 1:**
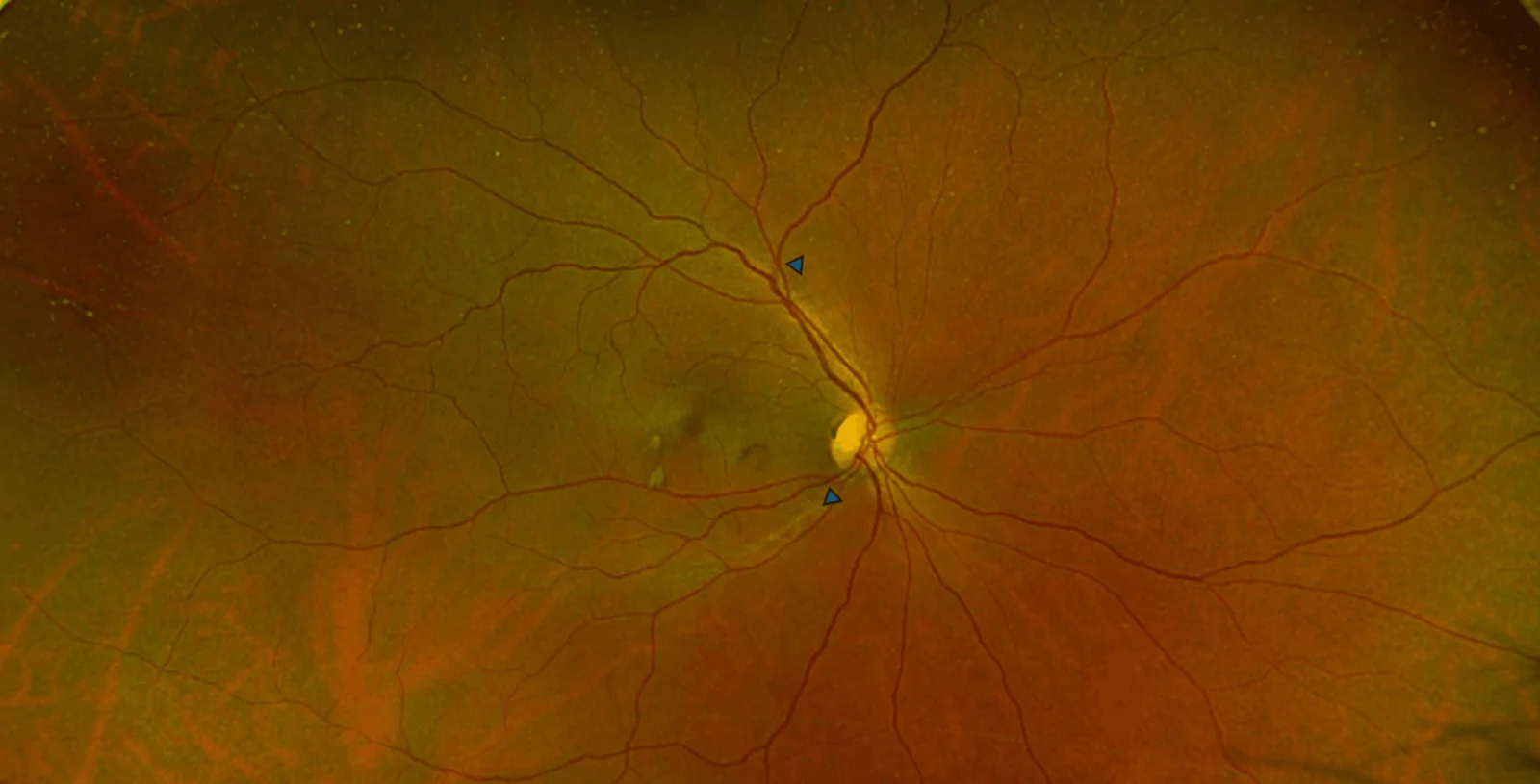
Fundus photo of the right eye. Fundus photo of the right eye showing arteriovenous nicking supertemporal and inferotemporal to the disc (blue arrowhead).

**Figure 2:**
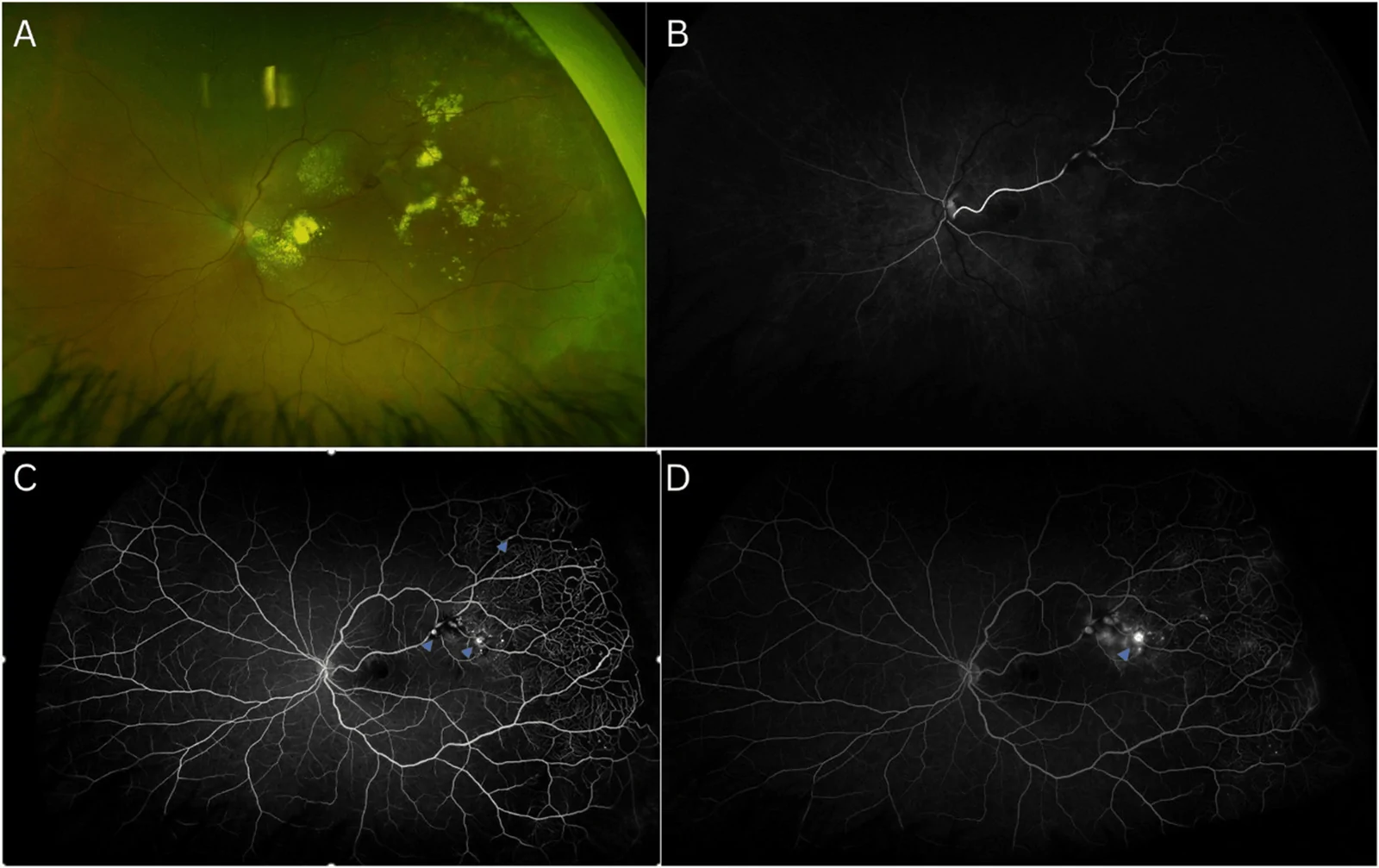
Wide field fundus imaging of the left eye. (A) Fundus photo of the left eye showing an anomalous cilioretinal artery coursing through the macula with three abnormal vascular outpouchings seen along its course, and multiple dense yellowish lipid deposits. (B) Early arterial phase fluorescein angiography reveals early filling of the cilioretinal artery arising from the temporal margin of the optic disc head. (C) Early recirculation phase of the fluorescein angiography showing three macroaneurysms (blue arrowhead). (D) Late recirculation phase showing leakage from a single macroaneurysm (blue arrowhead).

**Figure 3:**
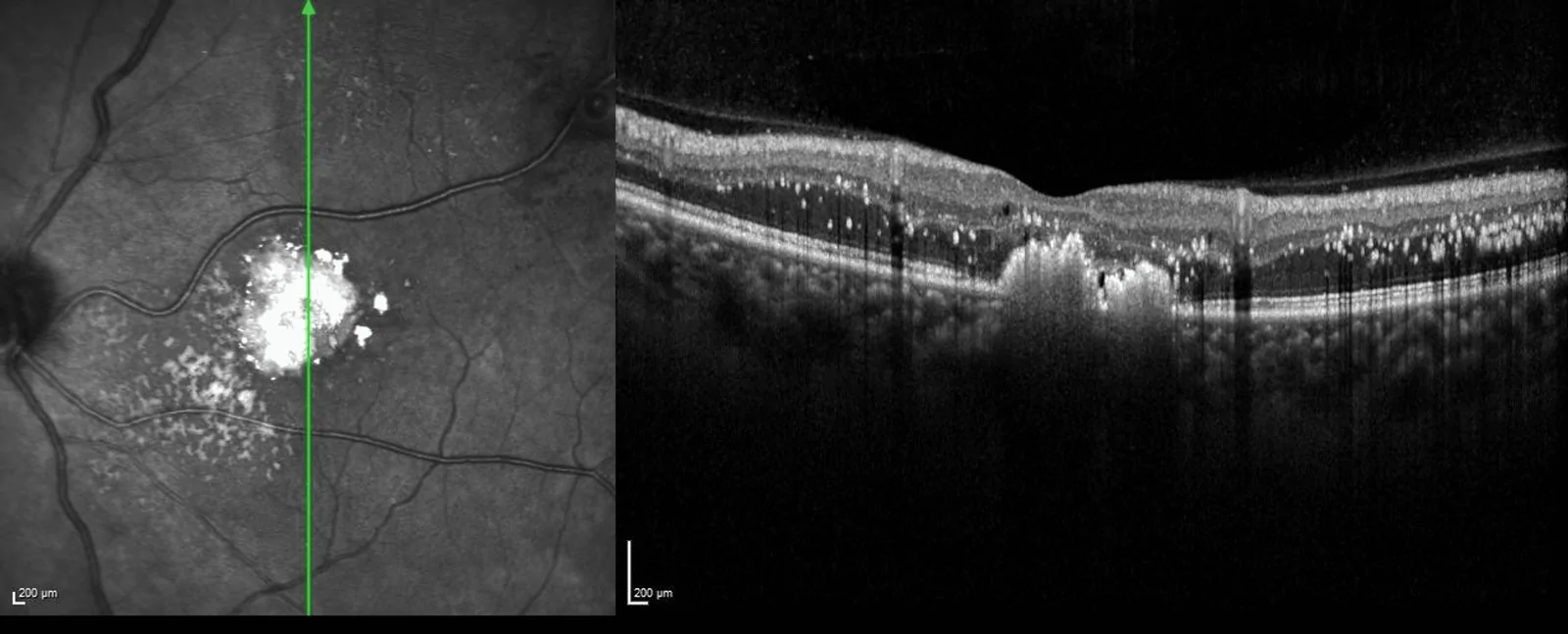
Macular optical coherence tomography of the left eye. Macular optical coherence tomography of the left eye showing mild intraretinal fluid with a hyperreflective lesion underneath the fovea as well as multiple hyperreflective dots at the outer retinal layers consistent with lipid.

The patient subsequently underwent focal laser photocoagulation treatment in the left eye for the leaking macroaneurysm, as well as peripheral scattered laser photocoagulation to areas of capillary non-perfusion due to concern for ischemia-driven vascular complications. Given the marked foveal exudation and intraretinal fluid, three monthly intravitreal bevacizumab injections (1.25 mg/0.05 mL) were administered as adjunctive therapy. The patient was lost to follow-up and then presented again to the retina clinic four years later, reporting no subjective improvement in vision. Her blood pressure measurement was normal as she established care with an internal medicine specialist, with whom she started antihypertensive medication. Her examination revealed the same best-corrected visual acuity of 20/30 in the right eye and 4/200 in the left eye. IOP and slit lamp examination were unchanged, with no new findings compared with her visit four years ago. DFE of the right eye remained the same as baseline. DFE of the left eye revealed macular exudation with foveal involvement, and a macroaneurysm that was regressing in size compared to the previous visit, in addition to peripheral temporal laser scars (Figure 4). Repeat FFA in the left eye indicated mild leakage from one of the macroaneurysms with stable appearance of peripheral retinal non-perfusion and absence of retinal neovascularization (Figure 4). Macular OCT indicated a retinal cyst with dense lipid maculopathy involving the fovea in the left eye (Figure 5). The patient received augmentation of scattered laser photocoagulation to the area with peripheral retinal non-perfusion. Focal laser photocoagulation of the leaking macroaneurysm and intravitreal aflibercept were offered to the patient after counseling on the risks and benefits. However, she declined further focal laser treatment and intravitreal injections because she had not experienced subjective visual improvement following prior treatment, likely due to persistent extensive lipid maculopathy despite previous laser therapy and three bevacizumab injections (Table 1).

**Figure 4:**
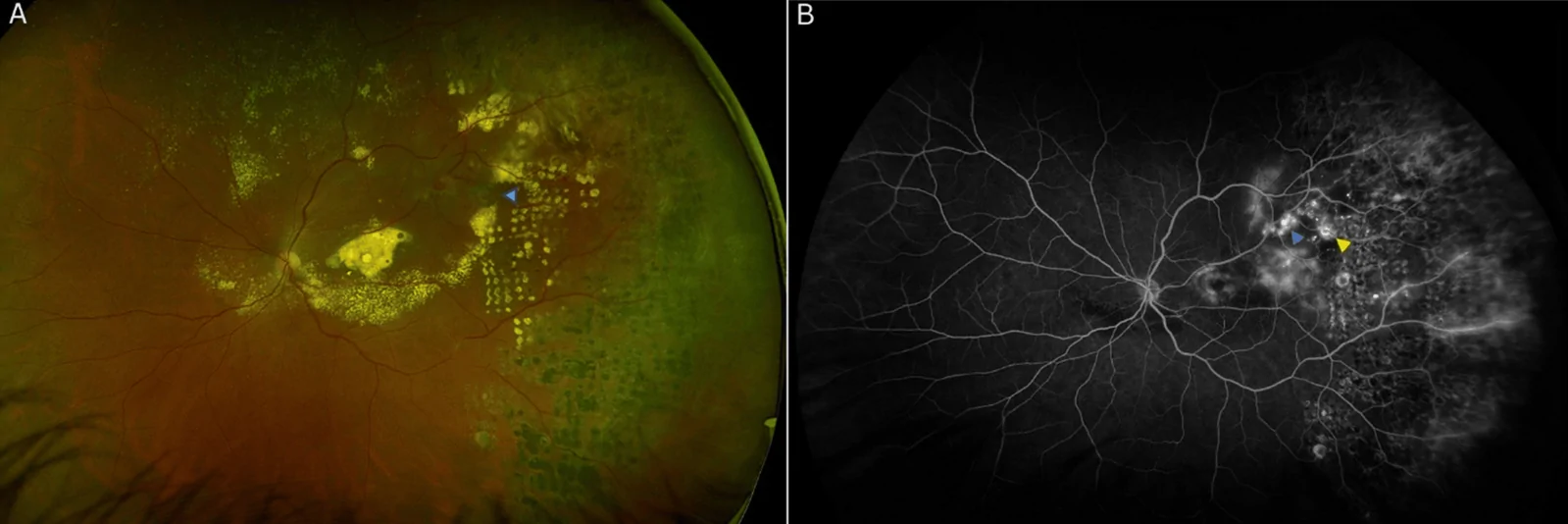
Wide-field imaging of the left eye. (A) Fundus photo of the left eye shows persistent lipid deposits at the macula, peripheral scatter laser photocoagulation scars, and a previously applied focal laser scar (blue arrowhead). (B) Late-phase fluorescein angiogram showing less active leakage from previously treated macroaneurysm (yellow arrowhead), and active leakage from the previously untreated macroaneurysm (blue arrowhead).

**Figure 5:**
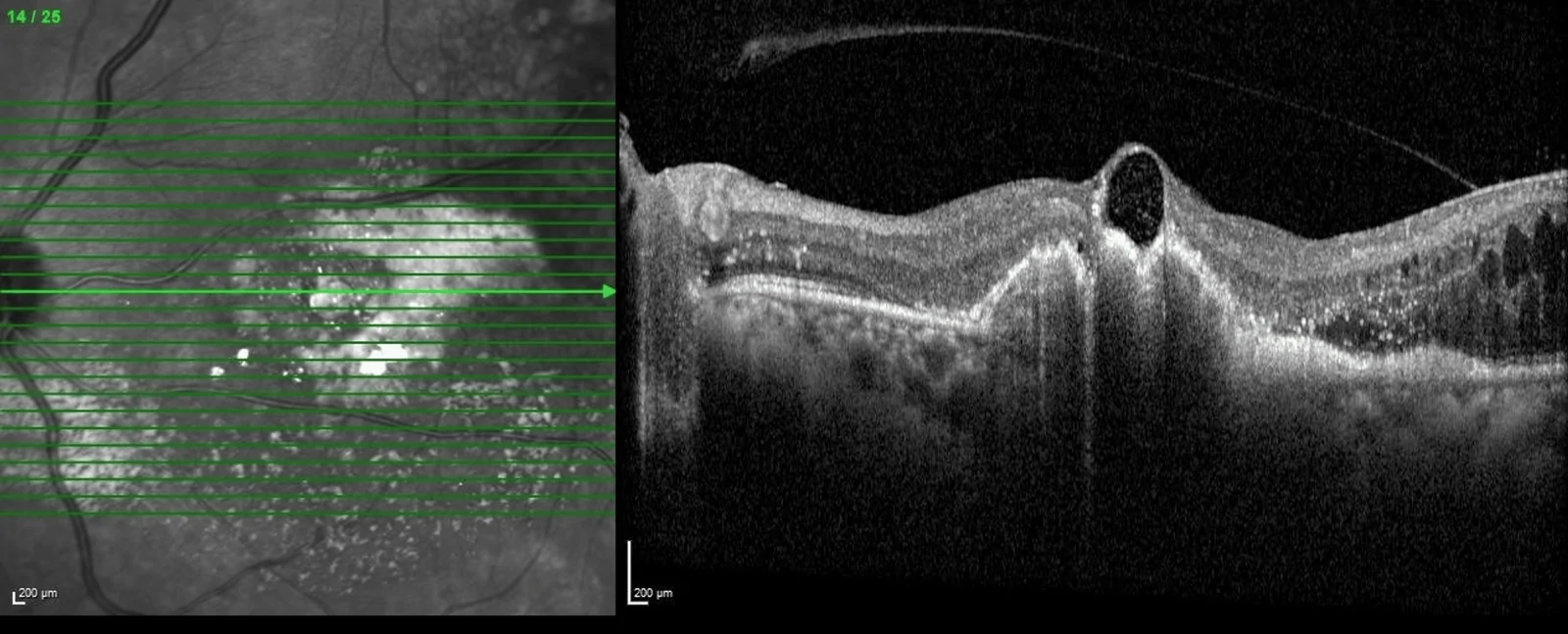
Macular optical coherence tomography of the left eye. Macular optical coherence tomography of the left eye showing dense subretinal hyperreflective material representing lipid maculopathy, with an overlying retinal cyst and intraretinal fluid.

**Table 1:** Timeline of clinical findings and management. BCVA = best-corrected visual acuity; BP = blood pressure; OD = right eye; OS = left eye; OU = both eyes

Time point	Findings/Investigations	Management
Initial Presentation	BCVA 20/30 OD and 4/200 OS. Marked fovea involving lipid exudation and intraretinal fluid OS. Multiple retinal arterial macroaneurysms along the anomalous cilioretinal artery. Temporal retinal capillary non-perfusion. BP: 180/110 mmHg	Focal laser to the actively leaking macroaneurysms, peripheral scatter laser to temporal non-perfusion, and three-monthly intravitreal bevacizumab injections as adjunctive therapy for the macular exudation
Lost to follow-up	No ophthalmic follow-up data available during this interval	No ophthalmic follow-up data available during this interval
Four years after the initial presentation	BCVA unchanged OU. Persistent foveal exudation and residual retinal cyst present with regressing cilioretinal artery macroaneurysms causing mild residual leakage. Stable peripheral non-perfusion of temporal retina. BP controlled on antihypertensive therapy	Scattered laser photocoagulation was augmented to the area with peripheral retinal non-perfusion. Focal laser photocoagulation of the leaking macroaneurysms and intravitreal aflibercept were offered to the patient after counseling on the risks and benefits. However, she declined given guarded prognosis

## Discussion

Retinal artery macroaneurysms are acquired fusiform or saccular vascular dilations that primarily involve the arterial tree in hypertensive and elderly women [7,10]. It has been proposed that atherosclerotic changes, including thickening of the vasculature and replacement of the contractile elements with collagen tissue, predispose to aneurysmal formation [11]. Although macroaneurysms are typically solitary and unilateral, in up to 20% of cases they are multiple, and in 10%, they are bilateral [12]. Impaired visual function in the setting of retinal arterial macroaneurysms is likely due to secondary multilayered hemorrhage, severe exudation, macular edema, or arterial occlusion [13,14]. Although observation is satisfactory for asymptomatic macroaneurysms, symptomatic lesions with significant macular involvement can be managed with focal laser photocoagulation, intravitreal injection of anti-vascular endothelial growth factor, or a combination of approaches depending on the clinical presentation [14,15].

The arterial macroaneurysms usually involve the retinal arteries rather than the ciliary arteries, such as the cilioretinal artery [8,9,16]. The hemodynamics and histopathological analysis of the two arteries were found to be similar, which cannot explain the disparity and predilection for retinal arteries. However, it has been hypothesized that preference in involvement of retinal arteries could be explained on an anatomical basis, including the latter being longer in association with AV crossings and vascular tortuosity, which may subject the retinal arteries to greater hemodynamic stress. The relative paucity of macroaneurysms involving the cilioretinal artery has long been attributed to its short course, lack of bifurcations and tortuosity, and absence of AV crossings [9]. In our patient, the unusually elongated course, extensive branching, tortuosity, and AV crossings of the cilioretinal artery were consistent with the hypothesis that vascular geometry may alter local hemodynamic stresses and potentially predispose to macroaneurysm formation. However, a definite causal relationship cannot be established from only a single case [9].

Congenital retinal macrovessels and Coats disease were considered in the differential diagnosis, but clinical and imaging features ruled them out in our patient. Congenital retinal macrovessels are large aberrant veins that cross the horizontal raphe within the posterior pole and are frequently associated with direct AV communications [17]. In our patient, the involved vessel was a cilioretinal artery with an unusually elongated peripheral course and extensive branching, with no evidence of AV malformation. Furthermore, the vascular anomalies in our case were characterized by several arterial macroaneurysms along the arterial course rather than a single large aberrant vessel. Similarly, although marked exudation and peripheral non-perfusion may superficially resemble Coats disease, in which telangiectatic vascular changes involve multiple retinal capillaries and small vessels within the affected sector, in our patient, every aneurysmal lesion was confined to the course and bifurcations of a single anomalous cilioretinal artery, with no angiographic evidence of diffuse retinal vascular telangiectasia beyond this arterial distribution [18]. The patient’s vascular findings and distribution pattern were therefore considered more consistent with numerous retinal arterial macroaneurysms in conjunction with hypertensive vasculopathy and an abnormal cilioretinal arterial configuration.

Chronic, previously unrecognized hypertension may have contributed to the vascular alterations observed in the present case. Arteriosclerotic changes due to hypertension, together with unusually elongated course and extensive branching of the cilioretinal artery, may have altered local blood-flow dynamics and mechanical stresses along the arterial wall, potentially predisposing to macroaneurysm formation [10]. The resulting aneurysmal dilations may have contributed to focal compression of adjacent retinal veins and impaired venous drainage, providing a possible mechanism for the peripheral retinal non-perfusion demonstrated on fluorescein angiography. Hence, we preferred to perform peripheral laser photocoagulation targeting the temporal retinal region to reduce the risk of further ischemia-driven retinal complications such as neovascularization.

Management of symptomatic retinal arterial macroaneurysms depends on the location and severity of the associated complications, including hemorrhages, exudation, and macular involvement. Focal laser photocoagulation can reduce persistent leakage, while intravitreal anti-vascular endothelial growth factor therapy has also been described as an adjunctive treatment in eyes with significant macular exudation and intraretinal fluid [14,19]. In our patient, focal laser was directed to the actively leaking macroaneurysm, whereas anti-vascular endothelial growth factor therapy was administered as an adjunct to address the marked macular fluid and exudation. Peripheral scatter photocoagulation was applied separately to the temporal area of capillary non-perfusion because of concern for ischemia-related vascular complications. Despite treatment, visual acuity remained unchanged, with persistent lipid exudation in the macular area associated with residual intraretinal fluid after the prolonged loss to follow-up. Although focal laser photocoagulation is the modality of choice for retinal arterial macroaneurysms, direct laser treatment of an aneurysm arising from a prominent cilioretinal artery requires extreme caution because of the risk of vascular compromise and retinal ischemia [19]. This consideration was relevant to our patient as the cilioretinal artery supplied a large retinal territory, including the macular region. 

## Conclusions

This case describes a rare occurrence of multiple retinal arterial macroaneurysms arising along an abnormally elongated cilioretinal artery with extensive peripheral branches. The unique vascular architecture, along with chronic hypertensive vasculopathy, may have contributed to localized hemodynamic alterations predisposing to macroaneurysm formation, although a causal relationship cannot be established from a single case. The concomitant peripheral retinal non-perfusion and significant exudation presented a clinical picture resembling Coats disease, underscoring the importance of rigorous multimodal imaging and vascular characterization in establishing the proper diagnosis. Furthermore, this case highlights the therapeutic challenges associated with direct laser photocoagulation of macroaneurysms arising from a prominent cilioretinal artery, given the risk of vascular compromise and macular ischemia. Further studies are needed to better characterize the pathogenesis, natural history, and optimal management of this rare vascular presentation. Recognition of such atypical vascular patterns is critical, especially in hypertensive patients, as early detection and individualized management can help avert vision-threatening complications.
